# Interleukin-27 in Tuberculosis: A Sheep in Wolf’s Clothing?

**DOI:** 10.3389/fimmu.2021.810602

**Published:** 2022-01-18

**Authors:** Kristina Ritter, Jasmin Rousseau, Christoph Hölscher

**Affiliations:** ^1^ Infection Immunology, Research Centre Borstel, Borstel, Germany; ^2^ German Centre for Infection Research (DZIF), Partner Site Hamburg-Borstel-Lübeck-Riems, Borstel, Germany

**Keywords:** cytokines, tuberculosis, IL-27, protection, pathology

## Abstract

In tuberculosis (TB), protective inflammatory immune responses and the pathological sequelae of chronic inflammation significantly depend on a timely balance of cytokine expression. In contrast to other anti-inflammatory cytokines, interleukin (IL)-27 has fundamental effects in experimental *Mycobacterium tuberculosis* (Mtb) infection: the absence of IL-27-mediated signalling promotes a better control of mycobacterial growth on the one hand side but also leads to a chronic hyperinflammation and immunopathology later during infection. Hence, in the context of novel host-directed therapeutic approaches and vaccination strategies for the management of TB, the timely restricted blockade of IL-27 signalling may represent an advanced treatment option. In contrast, administration of IL-27 itself may allow to treat the immunopathological consequences of chronic TB. In both cases, a better knowledge of the cell type-specific and kinetic effects of IL-27 after Mtb infection is essential. This review summarizes IL-27-mediated mechanisms affecting protection and immunopathology in TB and discusses possible therapeutic applications.

## Introduction

Tuberculosis (TB) is an infectious bacterial disease caused by *Mycobacterium tuberculosis* (Mtb) that has challenged humanity for millennia (1). The industrial revolution in the 18^th^ and 19^th^ century, provided the basis for TB to reach epidemic levels in North America and Europe (1, 2). Although it can be assumed that the genus *Mycobacterium* originated more than 150 million years ago (3) TB was until then a rather rare endemic disease (2). Albeit Benjamin Marten suspected the infectious origin of TB as early as 1720, it was not until 1882 that Robert Koch was able to isolate the bacillus and provide evidence of Mtb as causative agent for TB in laboratory animals (1, 4). Since then, the successful treatment of TB has been a central topic in medicine.

With the discovery of streptomycin as an effective remedy for TB in 1944, it quickly became clear that the administration of a single antibiotic alone leads to rapid development of resistance (5, 6). This first demonstration of the requirement of a multi drug therapy, led to the arrival of the modern era of anti-TB chemotherapy with the elaboration of the standardized first-line TB treatment regimens that are still used today (6–8). However, since treatment regimens were developed decades ago, therapy of TB has remained largely unchanged (9). Nevertheless, TB remains a major public health problem with estimated one-quarter of the world’s population infected by Mtb leading to 1.4 million deaths in 2019 and the need for improved treatment and vaccination strategies is urgent (10). Adjunctive host-directed therapies (HDT) modulating host immune pathways during antibiotic treatment are in the focus of TB research because such a type of treatment could shorten therapy time, increases efficacy and overall prevent the development of drug resistance (11, 12). However, only understanding of the pathophysiological molecular mechanisms underlying the interplay between Mtb and the host’s immune system will provide a rational basis for the development of more effective and optimized regimens to combat and prevent this ancient disease.

Mtb is transmitted from person-to-person by the inhalation of infectious aerosolized droplets exhaled by people with active TB (13, 14). The bacteria have developed strategies to avoid the elimination and can replicate within macrophages (MΦ). This induces the production of cytokines and chemokines by infected MΦ leading to the accumulation of infected MΦ, neutrophils, monocytes, and dendritic cells (DC) at the infection site resulting in the formation of a granuloma (13, 15, 16). Granulomas are complex structures that concentrate immunological responses, restrain Mtb and prevent dissemination to other organs. In response to Mtb infection, most infected individuals mount an immune response, that is only partially effective and rarely completely eradicate the bacteria (13). However, active TB develops when these initial granulomatous lesions lose their capability to contain mycobacterial replication.

In the regulation and orchestration of the inflammatory response against Mtb infection and the subsequent balance between protection and immunopathology cytokines play a key role (17). Especially, pro-inflammatory cytokines produced by T helper (Th)1 [e.g. interferon-gamma (IFNγ)] and Th17 [e.g. interleukin (IL)-17A] cells are considered to be the main effector cytokines mediating subsequent protective immune functions important for controlling Mtb infection (18, 19). However, although pro-inflammatory cytokines are necessary for host protection against Mtb, they can also promote the development of immune-mediated pathological consequences in the lung (20).

Especially, the extent of importance of IFNγ in the protection against TB is currently debated due to a lack of correlation between IFNγ-levels and the degree of protection (21–23). In fact, IFNγ-deficiency in the murine model results in higher bacterial loads leading to decreased survival in the course of Mtb infection, whereas overproduction of IFNγ is accompanied by increased lung pathology and as a consequence also fatal to the host (24–27).

Another pro-inflammatory cytokine that is also closely associated with protective and pathological functions in TB is IL-17A (28). After experimental infection with Mtb, IL-17A is involved in the formation and stability of granulomas by promoting neutrophil recruitment and chemokine release (29, 30). On the other hand, during chronic TB IL-17A-mediated extensive neutrophil recruitment and persistent inflammation are associated with tissue damage and immunopathology (28, 30).

In contrast to these pro-inflammatory cytokines, inhibitory and anti-inflammatory cytokines exhibit immune-limiting mechanisms in TB. First of all, IL-10 appears to be a very important anti-inflammatory cytokine, since several studies in different infectious diseases demonstrated that the absence of IL-10 results in stronger Th1 immune responses accompanied by improved pathogen clearance (31–34) but eventually also in accelerated and fatal hyper-inflammation (35, 36). However, after Mtb infection IL-10-deficiency has only a very limited impact on protection and no effect on immunopathology (34, 37). Only on a susceptible genetic background the absence of IL-10 was accompanied by improved effector mechanisms (38). But when overexpressed, IL-10 impaired protective immune responses with decreased levels of IFNγ and IL-17A, alternative MΦ activation and the establishment of chronic infection (34, 39–41).

Compared to IL-10, IL-27 appears to play a far more important role as an anti-inflammatory cytokine during experimental TB. Although initially reported as required for mediating the differentiation of Th1 cells (42–44), subsequent work revealed that IL-27 is involved in regulating Th1, Th2 and Th17 immune responses (45–47). In the context of Mtb, the lack of the IL-27 receptor-alpha (Rα) chain in experimental mice resulted in decreased bacterial loads associated with increased Th1 and Th17 immune responses and improved granuloma formation (30, 48). Although IL-27Rα-deficient (^-/-^) mice exhibited better control of Mtb, the detrimental higher production of pro-inflammatory cytokines resulted in a hyperinflammatory systemic immune response and accelerated death (48). In this regard, IL-27 represents a ‘double-edged sword’ as it mediates both protective and pathological immune responses to Mtb infection (30, 48). This review therefore considers the possible mechanisms underlying this central immunoregulatory function of IL-27 in TB and suggests possible aspects of therapeutic and preventive applications.

## IL-27 and the IL-27 Receptor

**Figure 1:**
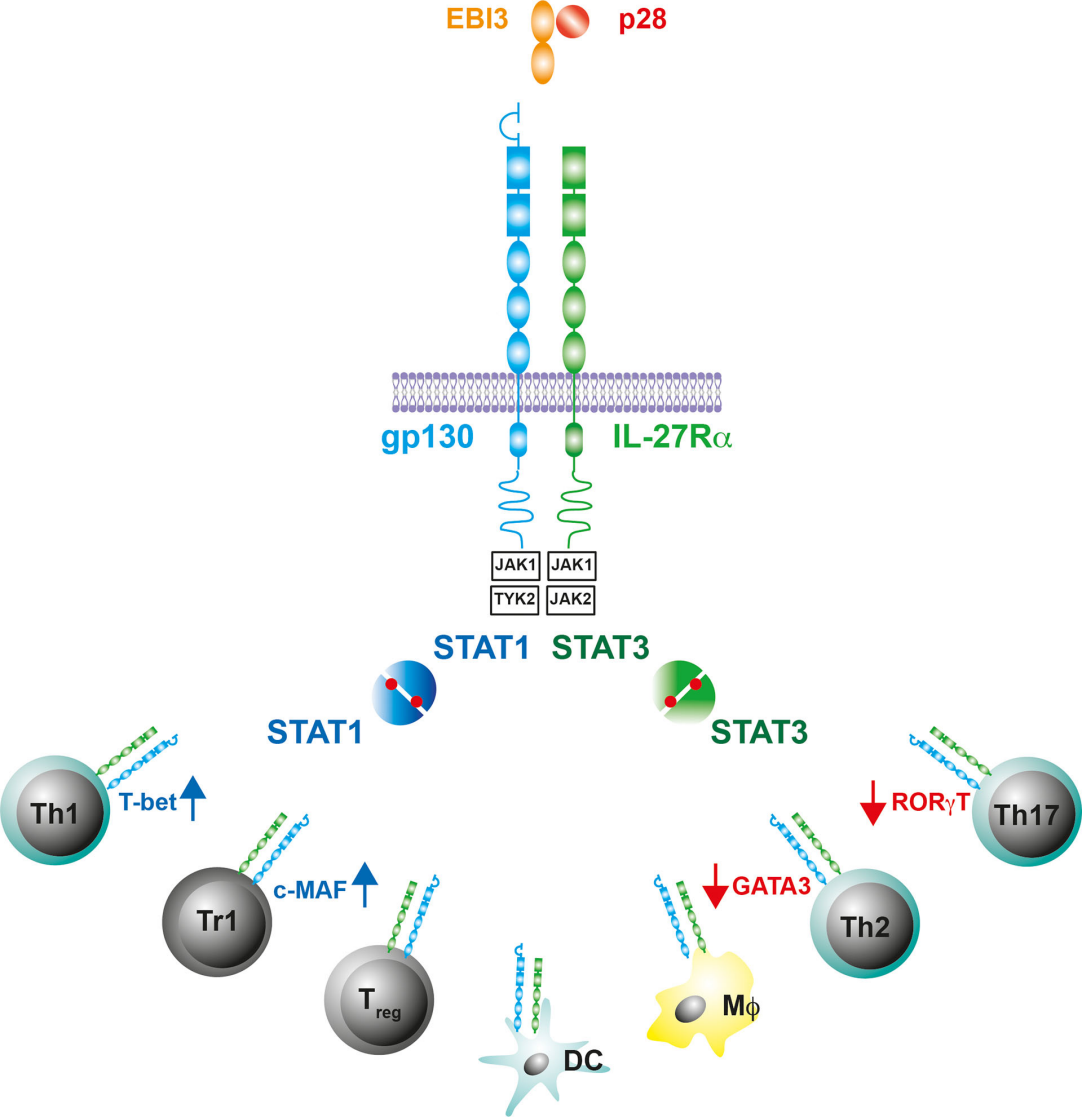
IL-27 and the IL-27R. The gp130 cytokine IL-27 is a heterodimeric cytokine comprising of a secreted receptor α-subunit and four-helix bundle protein (IL-27p28/EBI-3). The receptor complex for IL-27 contains gp130 and the IL-27R subunit-α, which is structurally similar to gp130. The intracellular domains of the receptor complex associate with the Janus kinases JAK1, JAK2 and Tyrosine kinase (TYK)2. IL-27-induced conformational changes of the receptor complex activate JAK1/TYK2 or JAK1/JAK2 and initiate a phosphorylation cascade that leads to the activation of signal transducer and activator of transcription (STAT)1 or STAT3, respectively. The IL-27R is predominantly expressed on T cells, Mϕ and DC where the Il-27R-mediated activation of STAT1/3 differentially affects the production of cell-type specific transcription factors.

IL-27, which was first described in 2002, is a member of the IL-6/IL-12 cytokine family (49–51). It forms a heterodimeric complex comprising of the α−subunit IL-27p28, a four-α-helix bundle protein, and the β subunit Epstein-Barr virus-induced gene 3 (EBI-3), which is structurally related to soluble receptors of this cytokine family (**Figure 1**). IL-27 is released by activated MΦ and DC (50, 52). Its signal transduction occurs through binding to a heterodimeric receptor complex consisting of the specific subunit IL-27Rα [formerly known also as WSX-1 or TCCR (T cell cytokine receptor)] and the ubiquitous gp-130 subunit (53)(**Figure 1**). The IL-27R is expressed on various immune cells but most notably on T cells, MΦ, and DC (**Figure 1**). The binding of IL-27 to its receptor complex leads to the phosphorylation of signal transducer and activator of transcription (STAT)1 and STAT3 *via* the activation of various kinases (**Figure 1**). According to the STAT1 pathway, IL-27 was initially assigned pro-inflammatory properties, as it induces T-box expressed in T cells (T-bet) and the expression of a functioning IL-12 receptor complex in naive CD4^+^ T cells whereby it instructs Th0 cells to develop a Th1 program (43, 46, 53, 54) (**Figure 1**). In contrast to this pro-inflammatory function during the early phase of a cell-mediated immune response, IL-27 mediates anti-inflammatory effects primarily *via* the phosphorylation of STAT3 (48, 55, 56). Basically, IL-27 can limit the strength and duration of Th1, Th2 and Th17 immune responses either directly or indirectly. It dampens overshooting Th1 cells and ameliorates corresponding type 1 inflammatory diseases (45, 48). Excessive Th2 and Th17 immune responses can be controlled by IL-27 through the downregulation of the transcription factors GATA3 (GATA binding protein 3) and retinoid-related orphan receptor gamma t (RORγt), respectively (46, 57, 58) (**Figure 1**). IL-27 can also indirectly regulate cell-mediated immune responses and excessive inflammatory reactions by promoting regulatory T_reg_ and Tr1 cells. It induces central molecules in T_reg_, which are essential for the function of these cells (59, 60). While the underlying mechanisms of this IL-27-dependent activation of T_reg_ are still largely unclear, it has been described for Tr1 cells that IL-27 mediates IL-10 production *via* the induction of the transcription factor c-MAF (61, 62) (**Figure 1**). But IL-27 does not only intervene in cell-mediated immune responses at the level of T lymphocytes. It can also inhibit the inflammatory reaction in activated MΦ and DC and thus indirectly control the development of Th1 and Th17 cells by reducing the release of IL-12, IL-6 and IL-23 (48, 63–65).

## The Dual Impact of IL-27 During TB

### During Experimental TB, IL-27 Limits Protective Immunity but Also Prevents From Immunopathology Caused by Excessive Inflammation

**Figure 2:**
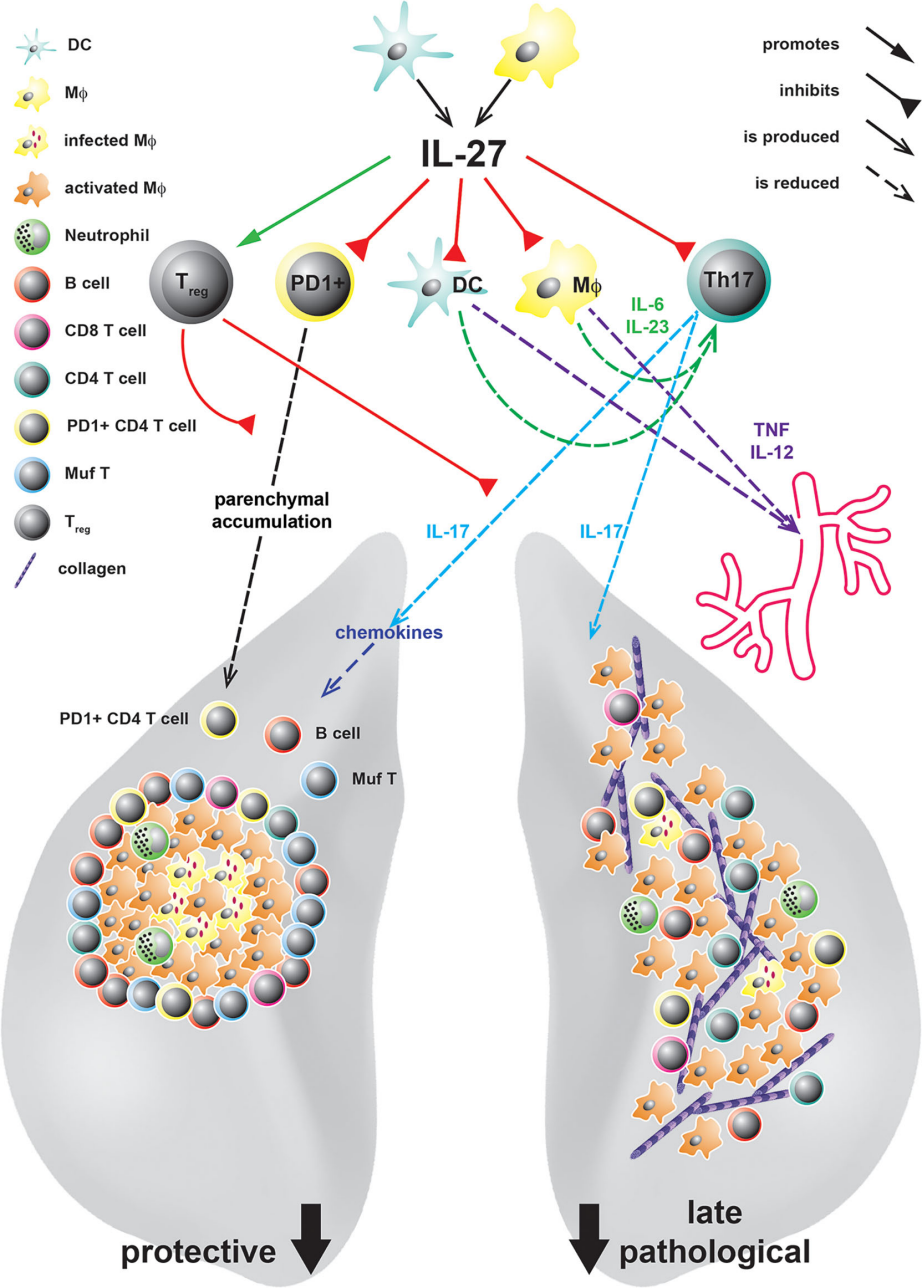
IL-27 in experimental TB and its protective and pathological immune responses at different states of Mtb infection. IL-27, produced by DC or MΦ in response to infection with Mtb, acts on various cell types. It controls the accumulation of PD1^+^ CD4 T cells in the lung parenchyma and limits the expansion of IL-17A-producing Th17 cells either directly or indirectly by increasing the numbers of T_reg_ or inhibiting the production of the Th17-driving cytokines IL-6 and IL-23 by DC and Mϕ. Thereby, defending mechanisms as infiltration of the lung tissue with PD^+^ CD4^+^ T cells, the chemokine-mediated formation of protective granulomas, the recruitment of B cells and IFNγ/TNF/IL-2-producing multifunctional (Muf) T cells as well as subsequent MΦ activation are diminished by IL-27. However, even if IL-27 suppresses these protective functions, during later states of infection it also controls lung-specific advanced inflammatory cell infiltration and interstitial collagen deposition but additionally systemic cytokine levels and eventually premature death.

Past *in vivo* studies on the role of IL-27 during TB focused on the analysis of mice with a constitutive deficiency in the private IL-27R domain IL-27Rα (IL-27Rα^-/-^) (30, 48, 66, 67). After infection with Mtb, these mice exhibit increased levels of the pro-inflammatory cytokines TNF and IL-12 followed by an enhanced activation of CD4^+^ T cells in the lung, which eventually culminates in reduced bacterial burdens (48). By reconstitution of lethally irradiated TCRβδ^-/-^ mice with IL-27Rα^-/-^ or intact bone marrow (BM), respectively, it was further demonstrated that T cells deficient in IL-27Rα are better able to control mycobacterial loads, indicating that the absence of IL-27Rα specifically on T cells accounts for the increased protection (67) (**Figure 2**). In the late phase of infection, however, IL-27Rα^-/-^ mice also suffer from uncontrolled chronic hyper-inflammatory immune responses accompanied by excessive systemic production of pro-inflammatory cytokines (48) (**Figure 2**). In consequence, in the later stages of Mtb infection, the animals develop secondary inflammatory symptoms. These include lung-specific advanced inflammatory cell infiltration accompanied with interstitial fibrosis, and deposition of cholesterol crystals (**Figure 2**) but also systemic pathological sequelae as splenomegaly, cachexia, and eventually shortened survival. Thus, IL-27 represents an important regulator during experimental TB with a dual function. On the one hand, the cytokine suppresses protective immunity in Mtb-infected mice, leading to a compromised mycobacterial containment (30, 48, 66), however, it also contains the pathological sequelae of chronic systemic inflammation (30, 48).

A hallmark of the host defence against mycobacterial infection constitutes the formation of granulomas (68). After infection with Mtb, alveolar MΦ express various inflammatory signals, such as TNF and the chemokines C-X-C motif ligands (CXCL)9 and 10, which mediate the recruitment of innate and adaptive immune cells to the sites of infection (69, 70). The resulting mature granuloma builds a microenvironment that brings effector T cells in close proximity to infected MΦ to optimally mediate mycobacterial killing (68). Importantly, also B cells aggregate within follicle-like structures around lung granulomas (71–74), which appear to contribute to granuloma organization and enhance protective immunity against TB (73). During experimental TB, IL-27 appears to have a negative impact on the architecture of lung granulomas and the localization of antigen-specific T cells (30, 67). Accordingly, Mtb-infected IL-27Rα^-/-^ mice at first develop a very efficient granulomatous response in the lung (30, 48) (**Figure 2**). In contrast to C57BL/6 mice, which show rather unstructured granulomatous lesions upon Mtb infection, granulomas in IL-27Rα^-/-^ mice are highly stratified containing a centre of infected MΦ surrounded by a pronounced rim of B and T lymphocytes (30). In contrast to these well-structured protective granulomas, however, moribund IL-27Rα^-/-^ mice in the late phase of chronic infection exhibit deconstruction of the granulomatous organization (48). As mentioned above, during this stage of infection, lungs show an accelerated tissue pathology connected to an overflowing pro-inflammatory cell infiltration, interstitial fibrosis and deposition of cholesterol crystals.

In addition to the histological differences, T cells in Mtb-infected IL-27Rα^-/-^ mice exhibit an altered phenotype and a modified cytokine expression pattern (30, 67). Accordingly, during chronic Mtb infection, lung CD4^+^ T cells in these mice are characterized by a decreased expression of the Th1 transcription factor T-bet and the killer cell lectin-like receptor G1 (KLRG1), while they maintain the phenotypic markers programmed death-1 (PD-1), CD69 and CD127 (67). In experimental TB, high expression levels of KLRG1 are claimed to identify a population of terminally differentiated effector CD4^+^ T cells (75). These cells are characterized by a high secretion of IFNγ along with a short life span and a low proliferative capacity. After vaccination against Mtb, the induction of antigen-specific KLRG1^-^ IL-2-producing CD4^+^ T cells, in contrast to KLRG1^+^ T cells, correlate with increased protection (76). PD1^+^ CD4^+^ T cells, on the other hand, represent an essential pool of activated self-renewing effector T cells which still bear the capacity to further differentiate into antigen-specific effector cells (75). Together, IL-27 seems to adversely affect the fitness of lung effector CD4^+^ T cells during experimental TB.

The presence of IL-27 during experimental TB furthermore affects the cytokine expression profile of T cells in the lung (30). Thus, so-called multifunctional CD4^+^ (Muf) T cells, which simultaneously express the cytokines IFNγ, TNF and IL-2, strongly accumulate in the lungs of Mtb-infected IL-27Rα^-/-^ mice (**Figure 2**). Muf T cells represent an effector T cell population with superior properties when compared to single cytokine-producing T cells (77–80). During Mtb infection, IFNγ and TNF appear to act synergistically to activate antimicrobial mechanisms in infected MΦ (79, 81, 82). Besides, the expression of IL-2 in CD4^+^ T cells enhances their competitiveness for long-term survival (83). When compared to single-cytokine-producing CD4^+^ T cells, Muf T cells have also been demonstrated to exhibit higher cytokine production levels on the single-cell level (30, 78, 80). In models of vaccination against Mtb, the induction of Muf T cells correlates with antimycobacterial protection (78, 80). Importantly, in contrast to the induction of Muf^+^ T cells, the overall frequencies of IFNγ-producing CD4^+^ T cells are not influenced by the absence of IL-27Rα-mediated signalling (30). By use of a C57BL/6 and IL-27Rα^-/-^ mixed BM chimera model, it has, on the other hand, been shown that the capacity to produce IL-2 is generally highly improved in IL-27Rα^-/-^ antigen-specific CD4^+^ T cells (67). In addition to the increased expression of IL-2, caspase activity in CD4^+^ T cells also appears to be markedly reduced in Mtb-infected IL-27Rα^-/-^ mice, pointing at lower susceptibility to cell death (67). Whereas T cell longevity therewith seems to be increased in the absence of IL-27-mediated signalling, conflicting data exist regarding the anti-proliferative activity of IL-27 in the context of Mtb infection (48, 67). In conclusion, the here described findings indicate that during experimental TB, CD4^+^ T cells in an IL-27Rα-deficient environment are characterized by their improved longevity and enhanced effector properties reasoned by the simultaneous expression of the cytokines IFNγ, TNF and IL-2.

The efficiency of antimycobacterial effector T cell responses is not only determined by their fitness and intrinsic qualities, but also by their localization near infectious pulmonary lesions. Upon Mtb infection, protective PD-1 and CXCR-3 expressing T cells are better able to infiltrate into the lung parenchyma, whereas IFNγ-expressing KLRG1^+^ terminally differentiated effector CD4^+^ T cells accumulate within lung blood vasculature (84, 85). In accordance with this finding, in Mtb-infected IL-27Rα^-/-^ mice the lower frequency of KLRG1^+^ CD69^-^ T-bet^hi^ CD4^+^ T cells results in a higher proportion of antigen-specific T cells within the lung parenchyma (67). Importantly, this parenchymal accumulation of CD4^+^T cells in the absence of IL-27Rα is also connected to an augmented colocalization of these cells with Mtb-antigen (67). Accordingly, Mtb-infected IL-27Rα^-/-^ mice exhibit enhanced MΦ activation reflected by increased gene expression levels of the antimicrobial molecules nitric oxide synthase-2 (NOS2) and IFNγ-dependent GTPase LRG-47 (48) (**Figure 2**). In the highly organized granulomas observable in IL-27Rα^-/-^ mice, T cells preferentially accumulate in the outer rim in proximity to the afore-mentioned B cell areas (30) (**Figure 2**). In this context, further investigation may reveal the relevance of B cell-mediated signals for the migratory capacity of protective CD4^+^ T cell within the Mtb-infected lung. Altogether, IL-27 seems to impede the ability of CD4^+^ T cells to assess the lung parenchyma to eventually hinder the direct association of effector T cells with infected phagocytes in lung lesions.

As mentioned before, IL-27 has been demonstrated to inhibit the development of Th17 immune responses in various mouse models of chronic inflammation (47, 58, 86, 87). Importantly, higher frequencies of IL-17A-expressing Th17 cells were also observed in the lungs of Mtb-infected IL-27Rα^-/-^ mice (30). By use of IL-27Rα^-/-^ mice with an additional deficiency for IL-17A, the impact of this increased production of IL- 17A in the absence of IL-27-mediated signalling was therefore further investigated. The obtained data revealed that IL-17A mediates to a large extent the enhanced protection in the IL-27Rα-deficient environment, but on the other hand, it also triggers the exacerbated immunopathology in Mtb-infected IL-27Rα^-/-^ mice (**Figure 2**). On the immunological level, IL-17A does not seem to exert any impact on the proportion of KLRG1^+^ CD4^+^ T cells, however, it mediates the accumulation of Muf T cells in the lungs of IL-27Rα^-/-^ mice (**Figure 2**). Moreover, the generation of highly stratified granulomas in the Mtb-infected IL-27Rα^-/-^ mice is strikingly dependent on IL-17A, which is not surprising given that the impact of IL-17A on TB granuloma formation has been described before (29). In this context, IL-17A also supports the expression of the T cell-attracting chemokines CXCL9, CXCL10 and CXCL13 in the lungs of the infected IL-27Rα^-/-^ mice (30) (**Figure 2**).

In conclusion, in the context of experimental TB, IL-27 functions as a key regulatory player with a dual impact on the outcome of Mtb infection. On the one hand, the cytokine hampers bacterial containment as well as the formation of highly organized protective granulomas in the lung, however, in the late phase of infection it also prevents from chronic hyper-inflammation and immunopathology (30, 48). It appears likely that IL-27-mediated – direct or indirect - effects on CD4^+^ T cells fundamentally affect protection during TB. The cytokine has been demonstrated to exert influence on both the intrinsic fitness as well as protective quality of Mtb-specific T cells subsets and the localization of these cells (30, 67). Importantly, the suppressive effect of IL-27 on the expression of the pro-inflammatory cytokine Il-17A also plays an central role, as, in the absence of IL-27Rα the cytokine affects both protective immunology but also immunopathology (30). However, as IL-27 constitutes a pleiotropic cytokine with IL-27R being expressed on various cell types, potential cell type-specific effects on other adaptive and myeloid cell subsets also need to be taken into consideration as discussed hereinafter. Future investigation may also put additional focus on the kinetic analysis of protective and deleterious effects mediated by IL-27. A better knowledge of both the cell type-specific and kinetic effects of IL-27 during Mtb infection is essential to evaluate potential therapeutic implementations for the management of human TB.

### From Mice to Men: IL-27 in Human TB and Potential Limitations of Experimental Studies

During human TB, increased levels of IL-27 are associated with active disease (67) and expression of both the IL-27p28 and EBI3 subunit was noticed in human TB granulomas (88). Moreover, TB pleural effusions also exhibit increased amounts of IL-27, pointing at a potential usage of the cytokine as a biomarker to differentiate between TB pleural effusions and other causes of pleural effusions (89, 90). Sources of IL-27 in TB pleural effusions appear to be monocytes, MΦ, B cells, both CD4^+^ and CD8^+^ T cells, NK/NKT cells as well as mesothelial cells (89). Notably, human TB pleural effusions were shown to contain a specific subset of terminally differentiated IL-27-secreting CD4^+^ T cells with a unique expression profile of pro-inflammatory cytokines, which may perform important functions in TB immunity through the interference with pleural mesothelial cells (91). Another study, however, identified gene polymorphisms associated with a pronounced reduction of IL-27 in individuals with pulmonary TB (92).

The enhanced expression of IL-27 during human active TB (67) underlines the relevance for investigating the role of the cytokine in the context of TB and is consistent with the improved mycobacterial containment in IL-27Rα^-/-^ mice (48, 66). Although Mtb infection studies in mice with a constitutive or conditional deficiency of IL-27Rα can provide crucial insights into mechanisms of action and functions mediated by IL-27, data thus obtained subject to several limitations. First of all, further cytokines have been demonstrated to signal through IL-27R. Accordingly, in B cells, the heterodimeric cytokine IL-35 signals through IL-27Rα and IL-12Rβ2 (93). IL-35 consists of EBI-3 and IL-12p35 and was demonstrated to have suppressive functions (94). Additionally, IL-27R, together with gp130 and IL-6Rα, forms a receptor for a heterodimer consisting of IL-27p28 and cytokine-like factor (CLF) (95). Furthermore, the absence of IL-27Rα may generally interfere with the balance between the cytokine subunits (67). Finally, other factors such as the route of exposure and the bacterial dose might have an impact on the experimental outcome. The combination of findings generated by experimental and human TB studies may therefore help to constitute a precise view of the IL-27-mediated immune mechanisms.

## Target Cells of Il-27 During TB: Who Are the Pivotal Players?

The broad expression of IL-27R on different cell types partially accounts for the complex regulatory functions IL-27 exerts in the context of infectious diseases (48, 63). As described before, *in vivo* studies figured out that protective immunity to Mtb infection in the IL-27R-deficient environment is substantially mediated through the intrinsic modulation and differential localization of effector CD4^+^ T cell populations (30, 48, 67). However, to obtain a more complete picture of the IL-27-mediated mechanisms affecting both protection and immunopathology in TB, it is important to additionally highlight potential specific effects of IL-27 on various IL-27R-expressing target cells (**Table 1**).

**Table 1:** Direct and indirect effects of IL-27-mediated signalling on different immune cell populations in the context of TB.

Cell type	Impact of IL-27^1^	Through suppression of IL-17A?^2^	Detailed description^1^
Th1	↓	–	Altered phenotype (enhanced numbers of KLRG1^+^ terminally differentiated CD4^+^ T cells) (67) Reduced accumulation of protective PD-1^+^ CD4^+^ T cells in the lung parenchyma (67)
Muf T	↓	+	Reduced frequency in the lung (30)
Th17	↓		Reduced frequency in the lung (30)
Tr1	↑	–	Enhanced frequency in the lung (30)
T_reg_	↑	–	Slightly enhanced frequency in the lung (30)
CD8^+^ T cells	↓	not described	Reduced frequency in the lung (48)
B cells	↓	+	Reduced localization at granulomatous lesions (30)
MΦ	↓	not described	Suppression of pro-inflammatory cytokine release (48) Inhibition of phagosomal acidification (96, 97) Inhibition of IFNγ-mediated autophagy (98)
neutrophils	↓	+	Reduced localization at granulomatous lesions (not connected to impaired protection, but potentially to reduced immunopathology) (30)

^1^Summary of findings obtained from in vivo studies in Mtb-infected IL-27Rα^-/-^ mice as well as in vitro studies in Mtb-infected murine and human MΦ; ^2^ Findings obtained from investigation of Mtb-infected IL-27Rα^-/-^/IL-17A^-/-^ mice (30); ↓ suppressive impact of IL-27-mediated signalling; ↑ promoting impact of IL-27-mediated signalling; – effect mediated by IL-27 in an IL-17A-independent manner; + effect mediated by IL-27 through suppressing IL-17A production in CD4^+^ T cells.

In contrast to the enhanced frequencies of Th1 and Th17 cells, numbers of IL-17A-expressing γδ T cells - which appear to represent a first line of defence during the early immune response to Mtb (99) - are not affected by the absence of IL-27Rα in the lungs of Mtb-infected mice (30). Therewith, it is likely that Mtb-specific Th17 cells alone account for the increased secretion of IL-17A in these mice, which is, in turn, connected to both the improved protection and deleterious immunopathology (30) (**Table 1**).

As part of the Mtb-specific CD4^+^ T cell immune response, IL-27 may - in addition to its regulatory influence on protective Th1 and Th17 cells - also exert influence on regulatory T cell subsets. As described before, IL-27 constitutes a potent promoter of IL-10-producing regulatory Tr1 cells (61, 62) and beneficially affects the functionality of T_reg_ (59, 60). In the context of experimental TB, T_reg_ accumulate at the infectious lesions, where they, in turn, may suppress protective immune responses against Mtb (100). Moreover, Mtb-specific T_reg_ delay the priming of effector CD4^+^ and CD8^+^ T cells in the pulmonary lymph node and eventually the onset of the adaptive immune response in the lung (101). Additionally, IL-10 was shown to attenuate protective antimycobacterial immune responses in both C57BL/6 and CBA/J mice (38, 40). Regarding the effect of IL-27-mediated signalling on regulatory T cell populations, reduced frequencies of Tr1 cells were found in the lungs of Mtb-infected IL-27Rα^-/-^ mice (30) (**Table 1**). In the chronic phase of experimental TB, the accumulation of T_reg_ also tends to be impaired in the absence of IL-27Rα (**Figure 2**, **Table 1**). Both effects, however, appear to occur independently of the elevated expression of IL-17A in IL-27Rα^-/-^ mice (**Table 1**). Importantly, a detailed functional analysis on the direct impact of IL-27 on T_reg_, has not been performed yet. To generate these data, Mtb-infected mice with a T_reg_-specific deficiency of IL-27Rα constitute a suitable experimental model.

Along with CD4^+^ T cells, CD8^+^ T cells appear to also contribute to containment of both the initial and long-term infection with Mtb (102). Especially, many assume that the simultaneous induction of both CD4^+^ and CD8^+^ T cells is fundamentally important in the context of efficient novel vaccination strategies against TB (103–105). In the absence of IL-27Rα, higher numbers of CD8^+^ T cells accumulate in the lungs of Mtb-infected mice (48) (**Figure 2**, **Table 1**). Additionally, these CD8^+^ T cells also exhibit enhanced expression of activation markers. Activated lung CD8^+^ T cells may therefore also be a part of the improved antimycobacterial immunity in IL-27Rα^-/-^ mice, eventually resulting in a more efficient containment of Mtb.

As mentioned before, in the absence of IL-27Rα, Mtb-infected lungs exhibit highly structured granulomas surrounded by a lymphocyte rim which predominantly contains clusters of B cells - an effect that is strikingly dependent on the expression of IL-17A (30) (**Figure 2**, **Table 1**). Although the role of these B cell clusters is not yet fully understood, it was demonstrated that in B cell-deficient mice reduced containment of mycobacterial loads correlates with an altered granulomatous response (73). Importantly, the accumulation of B cells in secondary lymphoid structures within in the peripheral rim of granulomas is also observable during human TB (106, 107).

The early establishment of Mtb infection is crucially determined by cells of the innate immune system (108, 109). Antigen presenting cells (APC), such as MΦ and DC, are able to initiate granuloma formation and adaptive immunity by the presentation of antigen and the secretion of pro-inflammatory cytokines, but on the other hand, they also create the primary niche for Mtb replication (108). Thereby, Mtb developed several mechanisms to escape from destruction within infected cells, including the blockade of phagosome maturation, regulation of autophagy and the restriction of antimicrobial peptide production. As IL-27R is known to be expressed on myeloid cells (42, 43, 48), IL-27 may also directly act on Mtb-infected MΦ (**Figure 2**, **Table 1**). Indeed, *in vitro* data demonstrate that IL-27Rα-mediated signalling directly affects the antimycobacterial activity in Mtb-infected murine and human MΦ (48, 97, 110). In murine IFNγ/LPS-stimulated or Mtb-infected peritoneal MΦ, the presence of IL-27Rα-mediated signalling suppresses the release of IL-12/23p40 and TNF, possibly through the concurrently observed induction of STAT3 phosphorylation (48, 111) (**Table 1**). Moreover, by incubation of human Mtb-infected MΦ with a soluble receptor to neutralize IL-27 (sIL27RA), it was shown that IL-27 antagonizes IL-18 activity by inhibiting the IL-18 receptor β-chain and blocking of NF-κB activation (110). IL-18, along with IFNγ and TNF, appears to play a critical role in the antimycobacterial activity of Mtb-infected MΦ when IL-27Rα-mediated signalling is blocked while IL-12 is supplied (112). Notably, upon stimulation of mouse BM-derived MΦ with LPS, IL-27 also triggers the production of the anti-inflammatory cytokine IL-10 *via* the activation of STAT1 and STAT3 (113). Together, these data indicate that IL-27 exerts differential impact on the secretion of pro- and anti-inflammatory cytokines from activated MΦ. The IL-27-dependent impaired release of Th1 and Th17-driving cytokines from Mtb-infected MΦ, in turn, compromises the development of protective T cell responses (**Figure 2**). In addition to these indirect MΦ-specific effects of IL-27 on protective immunity against Mtb, IL-27Rα-mediated signalling also appears to directly suppress antimicrobial effector mechanisms in the infected MΦ. Thus, in human MΦ, IL-27 interferes with the acidification of phagosomes and the subsequent process of phagosome-lysosome fusion by inhibiting the expression of vacuolar ATPase (V-ATPase) and lysosomal integrated membrane protein-1 (CD63) (96, 97) (**Table 1**). Additionally, the cytokine compromises the maturation of cathepsin D, which plays an important role for the degradation of mycobacteria and antigen processing within the cells (97, 114). It has also been described that IL-27 inhibits IFNγ-mediated autophagy through activation of the autophagy negative regulatory molecules mammalian Target of Rapamycin (mTOR) and Myeloid-cell leukaemia 1 (Mcl-1) to eventually avert the elimination of intracellular bacteria in Mtb-infected human MΦ (98) (**Table 1**). In conclusion, the outlined *in vitro* findings strongly suggest that IL-27 limits protective immunity against Mtb partially through its direct impact on infected MΦ. The relevance of a MΦ-specific deficiency of IL-27Rα on the outcome of Mtb infection, however, has not been experimentally tested yet. Instead, the effect of MΦ-specific lack of gp130 was investigated in the context of experimental TB (64). When compared to genetically intact control mice, MΦ-specific gp130-deficient mice express increased levels of Th17-driving cytokines and subsequently increased frequencies of IL-17A-producing cells yet do not exhibit any alteration in mycobacterial containment (64). In contrast, in the absence of gp130 on T cells, bacterial burdens actually appear to be moderately increased but Th17 immune responses were not significantly affected (115). When assessing data obtained in these mouse models, however, compensatory effects caused by other gp130-dependent cytokines, such as IL-6, must be taken into consideration. IL-27 also exerts immunosuppressive influence on human and murine DC (116), however, to our knowledge this effect was not yet investigated in the context of Mtb infection.

A well described function of IL-17A is the recruitment of neutrophils, which appears, in the context of experimental TB, to be associated with the early development of granulomas (117). Accordingly, in Mtb-infected IL-27Rα^-/-^ mice, IL-17A mediates the early accumulation of neutrophils in the centres of organized granulomas (30) (**Figure 2**, **Table 1**). Thereby, the anti-G-CSF induced reduction of neutrophils in the blood, however, does neither result in an altered granulomatous response nor in modified bacterial loads. This finding indicates that the protective role of IL-17A in the IL-27Rα-deficient environment is not mediated by its effect on the recruitment of neutrophils (**Table 1**). However, since high numbers of neutrophils are known to be associated with susceptibility in the chronic phase of TB (118, 119), the enhanced levels of neutrophils in the lungs of IL-27Rα^-/-^ mice may contribute to the emerging immunopathology during late infection.

Together, both *in vivo* and *in vitro* data indicate that upon infection with Mtb, IL-27-mediated signalling may exert direct impact on different cell subsets of both the innate and adaptive immune response. A broader knowledge of the cell type-specific effects of IL-27 could therewith help to uncouple protective and immunopathological mechanisms mediated by the cytokine. To this end, mouse models which exhibit a conditional deficiency of IL-27R within a specific cell type represent a beneficial tool.

## Concluding Remarks and Possible Applications

In the context of experimental TB, IL-27 turns out to be a sheep in wolf’s clothing: Even though the cytokine significantly hampers optimal containment of Mtb in the lung, the cytokine eventually protects from pathological sequelae of chronic hyper-inflammation in the late stage of infection (48).

*In vivo* mouse data provide a comprehensive picture of the IL-27-mediated effects on the structure of lung granulomas as well as on the localization and intrinsic quality of effector CD4^+^ T cell subsets during Mtb infection (30, 67). In this context, the IL-27-mediated suppression of pro-inflammatory Th17 cells appears to fulfil a major function (30). Accordingly, both the decreased bacterial burdens and the enhanced immunopathology in the absence of IL-27Rα are dependent on the expression of IL-17A. However, initial findings from *in vivo* and *in vitro* studies indicate that the pleiotropic cytokine IL-27 may also affect other innate and adaptive immune subsets during TB (30, 48, 96–98).

Although the role of IL-27 in TB is certainly complex, two possible strategies can be identified, of how a specific interference with IL-27-related immune mechanisms may improve novel therapeutic and preventive approaches. One the first hand, the improved control of mycobacterial growth in IL-27Rα^-/-^ mice indicates that the timely restricted blockade of IL-27 might constitute an interesting future option in the context of novel HDT or vaccination strategies. This approach is further substantiated by the observed up-regulation of IL-27 in patients with active TB (67). Notably, a naturally occurring soluble form of IL-27Rα (sIL-27RA) has been described, which inhibits the binding of IL-27 to its cell surface receptor (120). Treatment with recombinant sIL-27RA, moreover, was demonstrated to be beneficial during septic peritonitis (121). So far, however, the impact of a timely administration of this inhibitor during primary Mtb infection or vaccination on the outcome experimental TB has not been investigated. A second treatment option, in contrast, would emphasize to dampen the exacerbated inflammation in late phase of chronic TB by administration of IL-27 itself, as indicated by the present mouse experimental data (48). In this context it may at first be important to consider that, although the continuous treatment with immunomodulatory drugs, such as TNF-antagonists, is linked to an increased risk of reactivated Mtb infection in patients with latent TB (122), anti-inflammatory therapeutics against TB can have beneficial effects when administered in combination with antibiotics during adjunct therapy (123). A contrary indication may certainly be the observation that up-regulation of IL-27 has been connected to the occurrence of immune reconstitution inflammatory syndrome (IRIS), a phenomenon which is observed in TB patients coinfected with HIV (67, 124). However, it is not yet known if the cytokine exerts any functional impact on the development of this inflammatory disorder. Generally, further experimental and human research is needed to clarify a potential usage of Il-27 in the context of immunomodulatory HDT approaches against chronic TB.

Altogether, the key to a safe and efficient therapeutic intervention with IL-27-related immune mechanisms in the context of anti-TB management may be the spatiotemporal uncoupling of the protective and immunopathological effects mediated by the cytokine and, eventually, a very specific usage of therapeutics on this background. Basic research may help to gain a more precise knowledge of the cell type-specific and kinetic effects of IL-27 after Mtb infection.

## Author Contributions

KR, JR, and CH contributed to conception of this review. KR and CH wrote the first draft of the manuscript. KR, JR, and CH wrote sections of the manuscript. All authors contributed to manuscript revision, read, and approved the submitted version.

## Funding

This work was supported by the German Centre for Infection Research (DZIF), the Cluster of Excellence Inflammation-at-Interfaces (EXC306) and the DFG International Research Training Group (IRTG 1911).

## Conflict of Interest

The authors declare that the research was conducted in the absence of any commercial or financial relationships that could be construed as a potential conflict of interest.

## Publisher’s Note

All claims expressed in this article are solely those of the authors and do not necessarily represent those of their affiliated organizations, or those of the publisher, the editors and the reviewers. Any product that may be evaluated in this article, or claim that may be made by its manufacturer, is not guaranteed or endorsed by the publisher.
